# Efficiency of the implementation of cardiovascular risk management in primary care practices: an observational study

**DOI:** 10.1186/s13012-016-0434-2

**Published:** 2016-05-13

**Authors:** Eddy M. M. Adang, Anne Gerritsma, Elvira Nouwens, Jan van Lieshout, Michel Wensing

**Affiliations:** 1 Radboud Institute for Health Sciences, Department for Health Evidence (133), Radboud University Medical Centre, P.O. Box 9101, 6500 HB Nijmegen, The Netherlands; 2 Radboud Institute for Health Sciences, Department of IQ Healthcare, Radboud University Medical Centre, Nijmegen, The Netherlands

**Keywords:** Primary care, Cardiovascular risk management, Technical efficiency, Data envelopment analysis

## Abstract

**Background:**

This study aimed to document the variation in technical efficiency of primary care (PC) practices in delivering evidence-based cardiovascular risk management (CVRM) and to identify associated factors.

**Methods:**

This observational study was based on the follow-up measurements in a cluster randomized trial. Patients were recruited from 41 general practices in the Netherlands, involving 106 GPs and 1671 patients. Data on clinical performance were collected from patient records. The analysis focused on PC practices and used a two-stage data envelopment analysis (DEA) approach. Bias-corrected DEA technical efficiency scores for each PC practice were generated, followed by regression analysis with practice efficiency as outcomes and organizational features of general practice as predictors.

**Results:**

Not all PC practices delivered recommended CVRM with the same technical efficiency; a significant difference from the efficient frontier was found (*p* < .000; 95 % CI 1.018–1.041). The variation in technical efficiency between PC practices was associated with training practice status (*p* = .026). Whether CVRM clinical tasks were performed by a practice nurse or a GP did not influence technical efficiency in a statistical significant way neither did practice size.

**Conclusions:**

Technical efficiency in delivering evidence-based CVRM increased with having a training practice status. Nurse involvement and practice size showed no statistical impact.

## Background

Across the world, health-care systems experience challenges due to ageing populations and increases in chronic and lifestyle-related diseases [1] and rising health-care costs [2]. Many policymakers believe that a strong primary care is a crucial component of the health-care system [3]. Primary care can only fulfil this expectation, if evidence-based prevention and treatment is provided in all eligible patients at reasonable costs. This study focused on the delivery of evidence-based prevention of cardiovascular disease in primary care. Cardiovascular diseases (CVD) are common in the Netherlands; the number of CVD-related hospital admissions was almost 375,000 in 2012 [4]. Many programmes have focused on prevention of CVD and related disease, but still, they remain an important cause of mortality and morbidity [5]. Cardiovascular risk management (CVRM) is largely provided in primary care (PC) in the Netherlands. Although these efforts have resulted in improved cardiovascular prevention care over recent years, there is a substantial part of CVD patients at risk in the Netherland that do not receive CVRM as recommended by prevailing evidence-based guidelines [6]. Little is known about the variation across general practice organizations regarding the efficiency of delivering evidence-based CVRM. In case of improvement of CRVM, substantial health benefits can be gained, while containing costs of healthcare at the same time [7]. Decision makers (both clinicians and policymakers) would be supported by insight into the efficiency of implementation of evidence-based CVRM in PC, as well as knowledge of associated organizational factors that influence the efficiency.

This study aimed at determining the technical efficiency of PC practices delivering recommended CVRM for patients with established cardiovascular disease and especially aiming at identifying factors associated with the variation in efficiency. Here, technical efficiency is associated with the use of optimal procedures (i.e. optimal implementation of procedures) and was defined as the extent to which a PC practice delivers evidence-based CVRM in relation to its inputs in terms of medical labour. For this purpose, we used a two-stage data envelopment analysis (DEA) approach.

DEA originates from management science and operation research and has mostly been used for benchmarking efficiency of organizational units in, for example, banking. It is a nonparametric approach determining a piecewise linear efficiency frontier along the most efficient organizational or decision-making units (DMUs) by means of linear programming. Then, DEA compares all inefficient DMUs to the efficient frontier. The PC practices (DMUs) delivering recommended CVRM that lay on the efficient frontier can be regarded as best practices. As a benchmarking technique, the DEA approach has gained much attention since it was first proposed in the late 1970s by Charnes A, Cooper WW and Rhodes E [8]. Presently, there are several thousand recorded scientific contributions, some theoretical and some applied [9]. DEA has been widely applied within the health-care setting mostly with a focus on hospitals, nursing homes and physicians (see for example [10–15]). Other applications of DEA are within the context of dialysis centres [16] and health systems [17]. A recent systematic review by Pelone et al. [18] shows 39 DEA applications in PC. According to Pelone et al. [18], DEA has a number of features which make it an attractive tool for efficiency measurement of PC delivery: ‘it can handle effectively the existence of multiple primary care resources (inputs) and multiple health outcomes (outputs) in the transformation process. Furthermore, it does not require strong assumptions about the underlying technology linking the inputs to the outputs, and it measures efficiency in relative -instead of absolute- terms.’

## Methods

### Study population and data

This study was based on the follow-up measurements in a two-arm cluster randomized trial with a block design [NCT00791362], which was executed from September 2008 until September 2012 [19]. Patients were recruited from 41 general practices in the Netherlands, thereby involving 106 general practitioners. Only patients with established CVD, namely angina pectoris, acute myocardial infarction, transient ischemic attack (TIA), ischemic stroke, peripheral arterial disease, aortic aneurysm and other chronic ischemic heart diseases, were included from these PC practices. All these practices were enrolled in the national accreditation programme (NHG-Praktijkaccreditering) and used electronic medical records and International Classification of Primary Care codes (ICPC codes), a worldwide system to label conditions in primary care [19]. All measures used for the analysis were derived from patient records; for a detailed description of the variables, we refer to the published study protocol [19]. This study focused on the delivery of evidence-based prevention of cardiovascular disease in primary care, which covers monitoring, counselling and preventive medication. This study however did not show an effect on primary outcomes [20].

The present study uses data from the trial by Nouwens et al. [19], but it does not answer questions in the context of the initial trial. For instance, it lumps data from intervention and control groups for an observational study. So, the present study is a newly post hoc developed study.

### Data-analysis

The patient-based dataset was collapsed to make a dataset of PC practice delivering CVRM summary statistics. This led to PC practice average scores for both LDL cholesterol and systolic blood pressure. The analyses on the collapsed dataset consisted of a two-stage DEA approach. DEA as a method of technical efficiency analysis was chosen in this study because it can deal with multiple inputs and outputs and needs no assumptions about the distribution between outputs and inputs. DEA deals best with homogenous and independent units, which perform the same function, such as PC practices [21]. The first stage was the estimation of the technical efficiency of general practice organizations in delivering recommended CVRM, using bootstrapped DEA. The second stage comprised of a regression analysis where estimated bias-corrected efficiency scores (from the first stage) were regressed on a set of preselected explanatory and case-mix adjusting variables. For the DEA, 41 PC practices were used. Technical efficiency in this study reflects the ability of a practice organization to obtain maximal output from a given set of inputs. In this case, output is in terms of implementation of recommended CVRM and input in terms of labour consumption (in time) of health-care providers. This is consistent with Farrell (technical) efficiency [9]. For an input orientation, input-based Farrell efficiency scores (*F*) are ≤1, with 1 indicating technical efficiency. General practices with a score of 1 are on the efficient frontier and used as benchmark for the other practices. Scores <1 are considered less efficient. A score of, for example, .9 indicates that it is possible to save 10 % off all inputs and still produces the same outputs. An output-based Farrell efficiency score ($F\ge 1$), for example 1.2, suggests that output can be expanded by 20 % without spending additional resources. In our study, this means that the implementation of evidence-based CVRM in a PC practice with a Farrell efficiency score of, for example, 1.2 could be 20 % more successful without the consumption of extra health-care resources by benchmarking itself to one or a combination of PC practices on the efficient frontier. So, Farrell efficiency values provide information about relative improvement potential. In this study, the following assumptions for DEA analysis were made:

- The DEA model runs under the assumption of constant returns to scale (CRS) as we adhere a policymaker perspective and assume that the underlying technology is in a ‘steady state’.
- An output-oriented DEA model is performed to explore how each general practice could proportionally maximize its outputs given the inputs provided and move to a point on the efficient frontier.
- DEA analysis does not require trade-offs between the various types of outputs or subjective judgments. Under the assumption that all general practices were homogeneous in their production technology (CVRM) and assigning the same importance to the inputs and outputs, no weight restrictions were given in the model.
- DEA computes all the aforementioned values for each PC practice, taking into account its efficient peer PC practice(s) that use similar input–output ratios, but at a more efficient level.

#### Stage 1

##### Measures of the DEA: the production function

*The input variables*

Input is defined here as the average amount of labour time spent on a CVD patient within a period of 3 months, which was operationalized in terms of consultations. Labour in a PC practice can be performed by a GP, a practice nurse or a practice assistant.

The inputs of the primary care practices production function were:

- Average number of consultations with a GP
- Average number of consultations with a practice nurse
- Average number of consults with a practice assistant

For establishing the input variables, all consultations per patient with each specific health-care worker were counted within a period of 3 months. Only consultations which were related to cardiovascular diseases, diabetes mellitus and chronic obstructive pulmonary disease were included. Also, the type of contact was taken into account, namely three different kinds were distinguished: telephone consultation, normal consultation and home visit. These were, respectively, counted and weighted with a factor .5, 1 and 1.5, respectively [22].

*The output variables*

Output was defined in terms of implementation of evidence-based CVRM as defined by prevailing clinical practice guidelines. The outputs of the primary care practices production function were:

- Performance indicator, based on prescribing of recommended preventive medication
- LDL cholesterol
- Systolic blood pressure

The variables that were used to create the performance indicator are statin and anticoagulants prescription. Both medications need to be prescribed for nearly all included patients, according to the CVRM guideline [23]. The original variables (statin and anticoagulants prescription) are the basis of the performance indicator and have two categories: 0 ‘no’ and 1 ‘yes’. The scale of the composited measure (performance indicator) has two categories, namely 1 for ‘imperfect adherence to the guidelines’ (meaning none or one of the medications recommended by the guideline is prescribed) or 5 for ‘full adherence to the guidelines’ (meaning all of the medications recommended by the guideline are prescribed). The two categories (1 and 5) were chosen to reflect a 20 % relative reduction in the risk of cardiovascular events for full adherence to the guideline compared to imperfect adherence [24, 25].

Under the assumption of an output-oriented CRS DEA model that outputs should be increased, wrong conclusions will arise for LDL and SBP. Therefore, both variables were transformed. For both, a linear transformation was used of the form: *y*_desirable_ = − *y*_undesirable_ + *v* > 0 (−LDL plus 10; −SBP plus 300) [17, 26].

DEA measures efficiency relative to an estimate of the efficient frontier.

The difference between the DEA score and the efficient frontier is the relative (in)efficiency. This, however, neglects the fact that the sample of DMUs (PC practices) on which the DEA is performed underlays statistical noise and random error. In fact, the obtained efficiency scores are subject to uncertainty due to sampling variation. We therefore used a bootstrap DEA approach to estimate the bias-corrected efficiency scores applying 1000 bootstrap replications as proposed by Simar and Wilson [27]. We used the Benchmark package in *R*. This approach returns results directly as Farrell efficiency measures [9, 28].

#### Stage 2

##### Variables in the regression analyses

The dependent variable was the estimated bias-corrected technical efficiency score of general practices in providing evidence-based CVRM. Explanatory variables were:

- *Practice size*: The size of a PC practice has been found to influence efficiency [29].
- *Training practice:* These practices educate future GPs, and therefore, their patients can benefit from more attention and a culture that is orientated on learning and reflection.
- *Task CVRM practice nurse:* If a practice nurse performs CVRM clinical tasks instead of the GP, research shows that chronic care is similar or better although not necessarily less costly [30, 31].

Case-mix adjusting variables were:

- *Age:* The older the patient, the more complicated treatments they have.
- *Gender:* Men have a predisposition for cardiovascular disease.
- *Diabetes mellitus:* Co-morbidity might influence the efficiency of the CVRM care [32, 33]. Diabetes is included because structured care programmes for patients with diabetes have been implemented nationwide in the Netherlands. These structured care programmes for diabetes have similarity with the implementation of CVRM. PC practices that have these structured diabetes programmes are more likely to implement CVRM successful.
- *Socio-economic status:* Whether or not a practice is located in a disadvantaged neighbourhood (a neighbourhood with several infrastructural and societal shortcomings) can influence CVRM care.

The second stage DEA analysis was performed within the context of an assumed censoring data-generating process (CDGP) (data piled up at a censoring point (i.e. the efficient frontier)) and a fractional data-generating process (FDGP). Under the assumption of a CDGP, usually, the bias-corrected output-oriented DEA scores were regressed on explanatory variables using a Tobit regression. There is discussion that the efficiency scores are not generated by a censoring process but are fractional or proportional data [34]. Then, in the model considered by Simar and Wilson [27], a truncated regression provides for consistent estimation in the second stage [34]. Both regression models (Tobit and truncated) were performed using the statistical package STATA 13.1. The truncated regression was considered the base case, and these results are explicitly reported in the main text and Table 2.

## Results

In total, 41 PC practices were taken into analyses, representing 1671 cardiovascular patients. From the analysis, 4 patients were excluded due to missing values for the performance indicator. Therefore, information of, in total, 1667 patients were taken into analysis and used as basis for the aggregated data on PC practice level. Table 1 shows descriptive data on variables (practice size, training practice, CVRM tasks for the practice nurse, sex, age, diabetes, socio-economic status and DEA score) in the analysis. The mean DEA and bootstrapped DEA score were, respectively, 1.05 (5 % percentile 1; 95 % percentile 1.14) and 1.05 (5 % percentile 1.01; 95 % percentile 1.12). The DEA analysis showed that 15 PC practices (37 %) were on the efficient frontier and served as benchmarks for the other practices (Table 1). The results point to a significant difference from the efficient frontier (*p* < .000; 95 % CI 1.018–1.041).

**Table 1:** PC practice characteristics

Practice number	Practice size	Training practice	Tasks CVRM practice nurse	Mean age	Sex (% men)	Diabetes (% of patients with diabetes)	Socio-economic status^a^	DEA score^b^
1	2473	No	Yes	66.30	63.0	10.9	No	1.105
2	2460	Yes	Yes	70.93	60.0	15.6	No	1
3	7500	Yes	Yes	66.55	74.5	32.7	Yes	1.084
4	3230	No	Yes	68.46	78.4	32.4	No	1
5	13,000	Yes	Yes	71.04	72.5	24.9	No	1.089
6	2600	Yes	Yes	67.68	52.5	17.5	No	1.004
7	4600	Yes	Yes	73.55	64.5	22.6	Yes	1.057
8	12,700	Yes	Yes	68.79	61.8	17.6	No	1.026
9	3400	No	Yes	66.76	69.6	28.3	No	1.092
10	3000	No	Yes	64.44	47.2	16.7	No	1.059
11	5300	Yes	No	66.49	79.1	25.6	No	1.010
12	4400	Yes	Yes	68.43	73.5	6.0	No	1.035
13	6800	Yes	Yes	67.57	62.9	0	No	1.042
14	3500	Yes	Yes	66.27	63.6	0	No	1
15	3200	Yes	Yes	65.53	70.6	14.7	No	1
16	2650	Yes	Yes	70.31	61.9	21.4	No	1.047
17	2500	No	No	69.90	68.8	37.5	No	1
18	4100	Yes	Yes	72.47	64.4	4.4	No	1.078
19	2998	No	No	66.90	59.0	2.6	No	1.049
20	2245	Yes	Yes	61.40	80.0	33.3	Yes	1.005
21	2820	Yes	Yes	69.71	60.5	26.3	No	1
22	2900	Yes	No	65.00	71.9	18.8	No	1.025
23	2286	Yes	Yes	72.40	68.0	12.0	No	1
24	2475	Yes	Yes	66.29	45.7	31.4	No	1.012
25	1900	No	Yes	65.13	37.5	50.0	Yes	1.014
26	2600	Yes	No	69.32	38.7	35.5	No	1
27	2650	No	Yes	57.00	75.0	25.0	Yes	1
28	2640	No	Yes	75.00	57.6	15.2	No	1.036
29	3600	Yes	Yes	68.82	78.9	5.3	No	1
30	4900	Yes	No	67.52	73.9	13.0	No	1.000
31	2620	Yes	Yes	68.25	67.9	17.9	No	1.004
32	8000	Yes	Yes	69.84	64.9	16.2	No	1
33	2758	No	Yes	65.87	82.6	15.2	No	1.009
34	3018	Yes	Yes	67.21	70.6	32.4	No	1
35	2648	Yes	Yes	71.94	62.9	17.1	No	1
36	3400	Yes	Yes	69.71	58.5	17.1	No	1
37	2600	No	Yes	67.94	57.6	6.1	No	1.120
38	6550	Yes	Yes	67.64	66.7	24.0	Yes	1.111
39	1850	Yes	Yes	72.33	61.5	30.8	No	1.045
40	7000	Yes	Yes	66.94	94.4	8.3	No	1
41	2600	Yes	Yes	72.50	70.6	14.7	No	1.039

^a^Whether or not a practice is located in a disadvantaged neighbourhood

^b^Output-oriented Farrell technical efficiency scores

Truncated regression results are found in Table 2. Practice size showed no significant impact on the efficiency of PC practices with CVRM (*p* = .055). Being a training practice had a significant positive effect (negative coefficient) on the efficiency (*p* = .026). Nurse involvement showed no impact on technical efficiency (*p* = .280). Case-mix variable diabetes was found to have a significant positive effect on efficiency of PC practices with CVRM. The Tobit regression found similar results (being a training practice, *p* = .042).

**Table 2:** Results of truncated regression analysis

	Bootstrapped truncated regression (1000 replications)			
	Coefficient	Bootstrapped		*p* value
		95 % confidence interval		
Practice size	6.86e-06	−1.59e-07	.0000139	.055
CVRM task	.024	−.0192	.066	.280
Training practice	−.053	−.099	−.006	.026
Age	.004	−.002	.010	.198
Gender	.078	−.064	.221	.281
Diabetes	−.177	−.351	−.004	.046
Socio-economic status	.056	−.009	.121	.089

## Discussion

This study provided insight into the technical efficiency of delivering evidence-based CVRM and illustrates the relevance of economic analysis of implementation in health-care organizations.

The analysis showed that several general practice organizations regarding their technical efficiency in providing recommended CVRM deviated from the efficient frontier. This implies that not all practices provide evidence-based CVRM at optimal efficiency; in fact, 15 of 41 practices provided evidence-based CVRM at optimal efficiency. Increased technical efficiency (better implementation of evidence-based CVRM for the same resources) is related to training practice status. We did not find evidence for the impact of nurse involvement and practice size on technical efficiency.

In efficiency analysis, there are two important competing methods, stochastic frontier analysis (SFA) and DEA [9]. According to Bogetoft and Otto, both methods have their own strengths and weaknesses and differ from each other with regard to (1) the usage, SFA is used more often in econometric efficiency analyses and DEA more often in operation research efficiency analyses; (2) the functional form of the efficient frontier, it is imposed a priori as in SFA or it is obtained a posteriori from the sample observations in an empirical way as in DEA and (3) whether they follow a deterministic or a stochastic approach (i.e. the distance of a DMU or PC practice from the efficient frontier is entirely attributed to its inefficiency as in DEA or partly due to statistical noise and random error, in addition to its inefficiency as in SFA) [9, 18]. Our method expands conventional DEA in a stochastic DEA which combines the nice features of conventional DEA (like no assumptions on functional form) and stochastic (SFA) methods. Stochastic DEA makes it possible to use in an unbiased way the DEA efficiency score in a multivariate regression analysis that provides insight in which factors influence efficiency of PC practices [27].

Some limitations of this study need to be discussed. The variation of efficiency across PC practices can partly be explained by the number of practices in the DEA. Including more practices would more accurately identify the efficient frontier; however, more practices can also cause some exogenous impacts of no interest or beyond control affecting the results.

Despite these limitations and the complexities of the methodology, we believe that the study has high relevance. Implementation science has largely focused on effectiveness of implementation strategies, but their efficiency is equally important. An effective yet costly implementation programme is often of little practical value, although it might contribute to scientific knowledge. DEA is a useful methodological tool for exploring this, which adds to the methods for efficiency research (see for example Pelone et al. [18]). For cardiovascular risk management, we found that primary care practices provide (‘produce’) evidence-based care at different efficiency levels. This information could guide both professional development and negotiations about reimbursement of cardiovascular care. Efficient practices have a number of characteristics, or lack organizational characteristics that are often assumed to be crucial, as discussed below.

The size of general practices (that is, number of registered patients) has been found to have a number of consequences. Higher practice size was associated with less physician time per 1000 patients, which suggested potential efficiency gains assuming equal quality [29]. The latter was not confirmed by this study, but the underlying mechanisms are complex. It might be that our study was underpowered (on PC level) with regard to practice size (*p* = .055). Moreover, larger practices provide more structured chronic disease care [35] and may therefore reach larger numbers of eligible patients, albeit at the cost of lowered technical efficiency in those who are diagnosed. This might have neutralized the impact of practice on efficiency in our analysis.

The positive impact of training practice status on technical efficiency was clear, but the practical relevance of this finding may be limited as not all practices can become training practices. There may be hidden resource use by GP trainees (that is, clinical work that is provided without a cost), which were not considered in the analysis. However, it is possible that training practices are truly more efficient, because these practices are regularly being assessed and stimulated to improve their performance as precondition of keeping their status as training practice. The effect may also be based on the selection of specific professionals into training practices, for instance professionals with higher interest in organizational topics.

Previous analysis on a different data set showed that more involvement of practice nurses was associated with improved cardiovascular risk management [30]. We found no evidence that this goes hand in hand with increased technical efficiency in delivering evidence-based CVRM. This is consistent with rigorous evaluations on the involvement of nurses in clinical activities in primary care, which found overall little impact of nurse involvement on cost or efficiency of healthcare [22]. Previous studies suggested that lower salary costs of nurses are offset by longer consultations and lower threshold for test ordering as compared to GPs.

Although it is generally assumed that co-morbidity is associated with decreased efficiency, this study found that the presence of diabetes actually enhanced efficiency of health-care delivery structured diabetes care has shown to be effective [32, 33] and is very well established care in the Netherlands. It overlaps partly with CVRM care which explains why ‘diabetes’ positively contributes to the efficiency of PC practice applying CVRM.

## Conclusions

We are careful in providing strong recommendations for practice and policy on the basis of this study. Nevertheless, we showed that efficiency of providing evidence-based cardiovascular risk management can be quantified, which identified substantial differences between practices.

Considering the results as well as the emerging research evidence on PC practice organization, we conclude that practice size is associated with a range of outcomes. Larger practice size seems to have both positive and negative effects. Involving nurses in clinical and preventive activities has a number of advantages, but it seems to have little impact on efficiency. The positive impact of training status is consistent with other research: ideally, all practices should become training practices.
